# Unlocking the NF-κB Conundrum: Embracing Complexity to Achieve Specificity

**DOI:** 10.3390/biomedicines5030050

**Published:** 2017-08-22

**Authors:** Federica Begalli, Jason Bennett, Daria Capece, Daniela Verzella, Daniel D’Andrea, Laura Tornatore, Guido Franzoso

**Affiliations:** Centre for Cell Signalling and Inflammation, Department of Medicine, Imperial College London, London W12 0NN, UK; f.begalli@imperial.ac.uk (F.B.); j.bennett@imperial.ac.uk (J.B.); d.capece@imperial.ac.uk (D.C.); d.verzella@imperial.ac.uk (D.V.); d.dandrea@imperial.ac.uk (D.D.); l.tornatore@imperial.ac.uk (L.T.)

**Keywords:** nuclear factor κB, NF-κB inhibitors, cancer, IκB kinase, Gadd45β, ubiquitin

## Abstract

Transcription factors of the nuclear factor κB (NF-κB) family are central coordinating regulators of the host defence responses to stress, injury and infection. Aberrant NF-κB activation also contributes to the pathogenesis of some of the most common current threats to global human health, including chronic inflammatory diseases, autoimmune disorders, diabetes, vascular diseases and the majority of cancers. Accordingly, the NF-κB pathway is widely considered an attractive therapeutic target in a broad range of malignant and non-malignant diseases. Yet, despite the aggressive efforts by the pharmaceutical industry to develop a specific NF-κB inhibitor, none has been clinically approved, due to the dose-limiting toxicities associated with the global suppression of NF-κB. In this review, we summarise the main strategies historically adopted to therapeutically target the NF-κB pathway with an emphasis on oncology, and some of the emerging strategies and newer agents being developed to pharmacologically inhibit this pathway.

## 1. Introduction

Just over 30 years ago, Ranjan Sen and David Baltimore discovered a protein complex that bound to a conserved immunoglobulin regulatory DNA sequence in activated B lymphocytes and that they referred to as “Nuclear Factor binding to the κ-Light-chain-enhancer B site” or, in short, nuclear factor κB (NF-κB) [1]. Decades of research into this factor have subsequently elucidated the multiple mechanisms of action of the signalling pathways NF-κB embodies, its ubiquitous presence in tissues and the pivotal roles it plays in governing a myriad of physiological processes ranging from the regulation of the immune system and inflammatory response to the regulation of autophagy, senescence, cell survival, cell proliferation, and cell differentiation [2,3,4,5].

Considering the multitude of stimuli that are capable of inducing NF-κB and the vast spectrum of functions NF-κB plays in normal tissues, it is perhaps unsurprising that these key features of the NF-κB pathway are frequently hijacked by cancer [6]. Indeed, aberrant NF-κB activation is a hallmark of most human neoplasias, where it drives oncogenesis, disease recurrence and therapy resistance, largely by inducing a transcriptional programme that suppresses apoptosis of cancer cells and orchestrates the inflammatory reaction in the tumour microenvironment (TME) [7]. There is also evidence that NF-κB can promote tumour cell proliferation, local invasion, metastatic dissemination, metabolism reprogramming and the epithelial-mesenchymal transition (EMT), thereby contributing to essentially all hallmarks of cancer [8,9,10]. In certain cancer types, such as multiple myeloma, diffuse large B-cell lymphoma (DLBCL), Hodgkin’s lymphoma and glioblastoma multiforme (GBM), NF-κB is frequently stably activated by recurrent genetic alterations of upstream regulators of its pathway [6,11,12,13,14,15,16,17,18,19,20]. More commonly, however, constitutive NF-κB activation in cancer ensues from inflammatory stimuli and other cues emanating from the TME or oncogenic alterations lying outside the traditional NF-κB pathway, such as *RAS* and *PTEN* mutations. These mechanisms are often responsible for inducing stable NF-κB activation in various types of solid malignancy, including colorectal, pancreatic, ovarian and breast carcinoma [11]. Indeed, the wealth of available genetic, biochemical and clinical evidence provides a compelling rationale for therapeutically targeting NF-κB in a wide range of human malignancies. Nevertheless, developing a specific and clinically useful NF-κB inhibitor has proven a seemingly insurmountable problem. Historically, the challenge with conventional NF-κB-targeting strategies has been to achieve contextual, selective inhibition of the NF-κB pathogenetic activities, given the pleiotropic physiological functions and ubiquitous nature of the NF-κB pathway.

This review will examine the main strategies historically adopted to therapeutically target the NF-κB pathway in cancer, illustrating the principal classes of synthetic compounds and natural products that have been developed to inhibit oncogenic NF-κB signalling, and focusing on some of the more promising emerging approaches being developed to overcome the historical limitations of conventional NF-κB-targeting therapeutics. For a more general overview of the NF-κB pathway and its regulation and functions throughout the course of oncogenesis, we refer to the excellent reviews that have already extensively covered these topics [21,22,23,24,25,26,27,28,29].

## 2. The Nuclear Factor κB (NF-κB) Pathway

In mammals, NF-κB comprises a family of five proteins, namely RelA/p65, RelB, p50/NF-κB1 (p105), p52/NF-κB2 (p100), and c-Rel, which form virtually all possible combinations of homo- and hetero-dimeric NF-κB complexes [5,30]. The members of this family are characterised by the presence of a highly conserved 300-amino acid N-terminal region known as the Rel-homology domain (RHD), which is responsible for the dimerization, DNA binding and nuclear translocation of NF-κB subunits, as well as their interaction with IκB regulatory proteins [4]. In resting cells, NF-κB complexes are normally held inactive in the cytoplasm by binding to members of the IκB family of proteins, including IκBα, IκBβ and IκBɛ. These proteins all contain a so-called ankyrin repeat domain (ARD), which interacts with NF-κB dimers and blocks their nuclear import by masking their nuclear localization signals (NLS) [4]. IκB proteins can also prevent nuclear NF-κB complexes from binding to DNA and can shuttle them out of the nucleus by means of their nuclear export signal (NES) [4]. The C-termini of the p105 and p100 precursor proteins also contain IκB-like ankyrin repeats, which must be degraded in order to generate the mature p50 and p52 subunits, respectively [8,28].

NF-κB can be activated from these latent cytoplasmic pools in response to a large variety of stimuli capable of causing the phosphorylation of IκB proteins on conserved serine residues by the IκB kinase (IKK) complex [31,32]. The site-specific IκB phosphorylation by IKK in turn creates a destruction motif, which is recognised by the SKP1-Cullin 1-F-box protein (SCF) E3 ubiquitin-protein ligase complex, SCF^βTrCP^, comprising the core subunits, SKP1 and Cullin 1 (CUL1), the RING component, RING-box protein 1 (RBX1; also known as ROC1/HRT1), and the F-box protein, β-transducin repeat-containing protein (βTrCP), in conjunction with a member of the Ubc4/5 family of E2 ubiquitin-conjugating enzymes, leading to the K48-linked polyubiquitination of IκBs at conserved lysine residues and their subsequent proteolytic degradation by the 26S proteasome [4,21,25,26,33,34,35,36,37,38].

Following the removal of IκBs, the released NF-κB complexes are free to enter the nucleus [4], where they bind to distinctive decameric DNA elements, known as κB sites, and regulate transcription of a diverse array of genes, encoding numerous inflammatory mediators, immunoregulators, apoptosis inhibitors, developmental factors and other genes responsible for moulding the host defence responses to stress, injury and infection [4,5]. Notably, the outcome of NF-κB activation is the induction of diverse and tightly controlled transcriptional programmes, which exhibit a wide degree of tissue- and context-specificity. Precisely how ubiquitous NF-κB complexes achieve this transcriptional diversity in tissues is not completely understood, but appears to hinge at least in part upon the specific composition of the NF-κB dimers being activated, their post-translational modification state, their interaction with other transcription factor pathways, and the specific configuration of the chromatin [5].

### 2.1. The Canonical NF-κB Pathway

**Figure 1:**
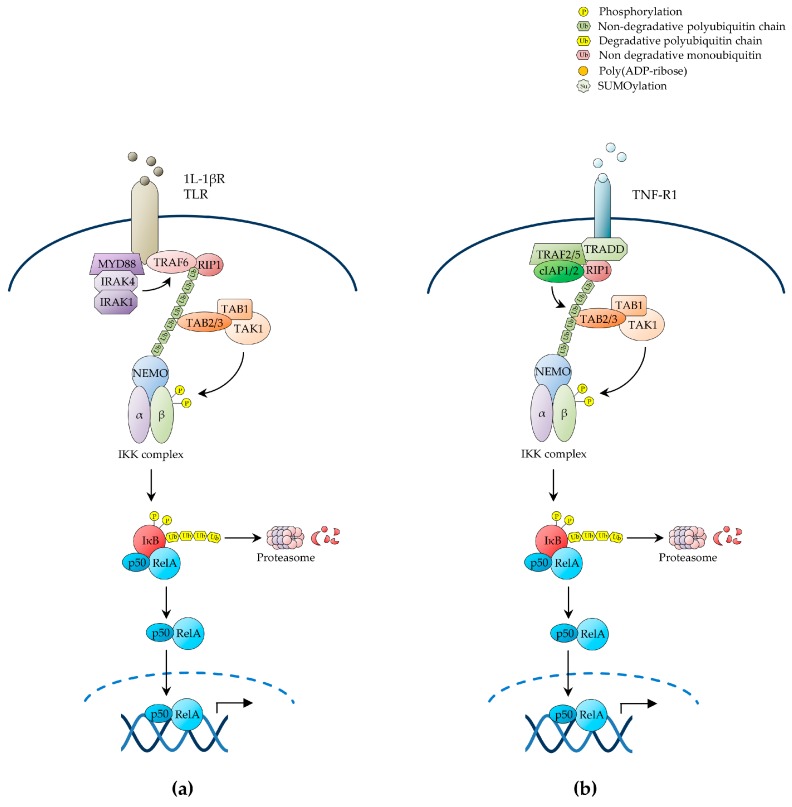

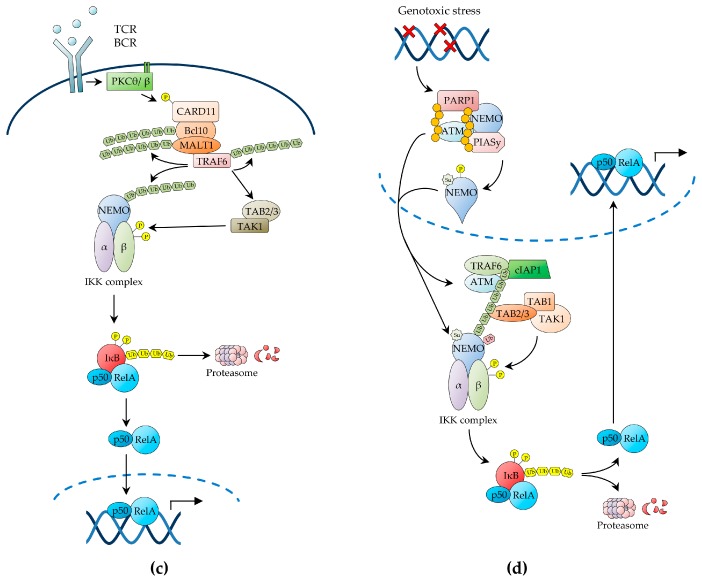
The canonical NF-κB pathway. Schematic representation of the three main upstream pathways of canonical NF-κB activation through: (**a**) IL-1βR and TLR receptors; (**b**) TNF-R1 receptor; (**c**) the antigen receptors, in B and T cells; (**d**) genotoxic stress. As results of the exposure to activating stimuli, NEMO is recruited to the respective proximal signalling complexes, bound to the receptors, through non-degradative polyubiquitin chains. The recruitment of the IKK complex via NEMO then allows the TAK1 kinase to activate IKKβ by the phosphorylation on specific T-loop serine residues, leading to IκBs’ phosphorylation and degradation, thereby freeing the active NF-κB dimers. NF-κB, nuclear factor κB; TLR, toll-like receptor; NEMO, NF-κB essential modulator; IKK, IκB kinase; TAK1, Transforming growth factor β-activated kinase 1.

The pathways of NF-κB activation are broadly classified as the canonical or classical pathway and the non-canonical or alternative pathway, depending upon the nature of the IKK complexes and IκB proteins involved. In the canonical NF-κB pathway, exposure to inflammatory cytokines such as tumour necrosis factor-α (TNF-α) and interleukin 1β (IL-1β), antigen or other immune signals, and microbial products such as pathogen-associated molecular patterns (PAMPs), leads to the rapid activation of an IKK complex typically comprising the two homologous catalytic subunits, IKKα and IKKβ, and the regulatory scaffold subunit, IKKγ (also known as NEMO) [32]. Upon engagement of their cognate receptors, these ligands trigger a signalling cascade that culminates with the K63-linked polyubiquitination of IKKγ/NEMO [39], a reaction that, unlike K48-linked ubiquitination, does not target proteins for degradation by the proteasome, but rather causes post-translational modifications, which enable IKKγ/NEMO to interact with other signalling effectors, leading to the downstream activation of IKKβ by site-specific phosphorylation on serine residues, S177 and S181, within the activation loop (Figure 1) [4,21,27,34,38,40,41].

The main physiological function of canonical IKK activation is to enable the IKKβ-mediated phosphorylation of IκBα on S32 and S36, thereby targeting the inhibitor for SCF^βTrCP^-dependent K48-linked ubiquitination at conserved lysine residues, K21 and K22, and subsequent proteolysis by the proteasome [8,34]. Biochemical and genetic studies have shown that IKKβ is both necessary and sufficient for IκBα phosphorylation through a mechanism that strictly depends upon IKKγ/NEMO. By contrast, IKKα is not involved in IκBα phosphorylation, but rather contributes to canonical NF-κB-mediated transcriptional responses by directly modulating the activity of NF-κB subunits, histones, transcriptional co-activators/co-repressors, and other non-IκB substrates [21,28]. While both IKKα and IKKβ have the ability to phosphorylate NF-κB subunits, such as RelA and c-Rel, and engage in crosstalk with other signalling pathways, such as the mammalian target of rapamycin (mTOR) and mitogen-activated protein kinase (MAPK) pathways, only IKKα is capable of accumulating in the nucleus and phosphorylating nuclear substrates, such as chromatin components [21,23,42,43].

Conversely, in addition to targeting IκBα, activated IKKβ, but not IKKα, phosphorylates the other canonical IκB proteins, IκBβ and IκBɛ, which, like IκBα, contain a conserved N-terminal signal-responsive motif, comprising two serine residues serving as target sites for IKKβ-mediated phosphorylation and one or two lysine residues that are targeted for inducible K48-linked ubiquitination by SCF^βTrCP^ and Ubc4/5-family E2 ligases [34]. Notably, whilst upon stimulation, IκBα is subject to rapid proteolytic degradation and resynthesis, the signal-induced proteolysis and resynthesis of IκBβ and IκBɛ occur with characteristically delayed kinetics [34]. Indeed, canonical IκB proteins are also functionally distinct in other important ways. For instance, unlike IκBα, nuclear IκBβ can associate with DNA-bound NF-κB complexes at specific promoter κB sites to prolong transcription of inflammatory genes, such as TNF-α, in response to bacterial lipopolysaccharide (LPS) [44]. Moreover, the expression and function of IκBɛ appears to be distinctively restricted to the haematopoietic lineage [4]. Irrespective of the mechanisms involved in the regulation and function of different IκBs, the primary outcome of canonical NF-κB activation, which is the predominant form of NF-κB signalling, is the release of RelA- and c-Rel-containing NF-κB dimers, the most ubiquitous and abundant of which is the RelA/p50 heterodimer [4,8,21,25,29,45]. Upon nuclear translocation, these dimers bind to DNA to coordinate the expression of inducible transcriptional programmes responsible for orchestrating immune and inflammatory responses, cell survival programmes and other host defence mechanisms, through a process that is characterised as being rapid and self-limiting [4,8,21,25,29,45].

#### 2.1.1. NF-κB Activation by IL-1β Receptor (IL-1βR) and Pattern Recognition Receptors (PRRs)

Multiple upstream signalling pathways, including those evoked by cell-surface receptors such as IL-1β receptor (IL-1βR), toll-like receptors (TLRs), TNF receptor 1 (TNF-R1) and T-cell receptor (TCR), cytoplasmic receptors such as nucleotide-binding oligomerisation domain (NOD)-like and RIG-I-like receptors, and stress signals such as genotoxic stress, converge on the IKK complex to induce canonical NF-κB activation [21]. Once activated, these receptors and their associated protein complexes coalesce into intracellular signalling networks that utilise adaptor protein interactions, protein phosphorylation, non-degradative ubiquitination and other signal-transducing mechanisms as means to attain the downstream activation of IKK. These signalling processes hinge on the signal-induced proximity and correct positioning of diverse multimeric units, and involve common signalling intermediates, such as TNF receptor-associated factor (TRAF)-family ubiquitin E3 ligases and the IKK kinase, transforming growth factor β-activated kinase 1 (TAK1) (Figure 1a) [21,24,27].

In the case of IL-1βR and TLRs, cognate ligand recognition triggers the recruitment to the receptor of the adaptor protein, myeloid differentiation primary response protein 88 (MYD88), and the protein kinases, interleukin-1 receptor-associated kinase (IRAK)1 and IRAK4, which in turn recruit TRAF6, thereby enabling it to oligomerise and manifest its ubiquitin E3 ligase activity [21,23,24,46]. Activated TRAF6 then catalyses its own K63-polyubiquitination as well as the K63-polyubiquitination of its target substrates, including IKKγ/NEMO and IRAK1, by operating in conjunction with a ubiquitin E2 ligase complex comprising Ubc13 and the Ubc-like protein, Uev1A [21,24,47]. The polyubiquitin chains of IRAK1 in turn form a binding scaffold that interacts, on the one hand, with the IKKγ/NEMO ubiquitin-binding domain (UBD) to recruit the IKK complex and, on the other hand, with the UBD of the TAK1-associated adaptor proteins, TAB2/3, to recruit the TAK1 kinase complex, also comprising TAB1, thereby enabling TAK1 to phosphorylate IKKβ on its T-loop serine residues, S177 and S181, resulting in the activation of IKK (Figure 1a) [34,48,49]. The MAPKKK, MEKK3, has also been shown to serve as an IKK kinase downstream of IL-1βR and TLRs, as well as of TNF-R1 [21,24].

The innate immunity pathways of NF-κB activation initiated by the engagement of intracellular NOD-like receptors (NLRs) and RIG-I-like receptors (RLRs) share an overall similar organisation and common signalling intermediates, such as TRAF proteins, with the NF-κB activation pathways triggered by IL-1βR or TLR stimulation [35]. NLRs are evolutionarily conserved cytoplasmic receptors activated in response to PAMPs, such as intracellular bacterial peptidoglycans, and damage-associated molecular patterns (DAMPs) such as cellular stress products [35]. Upon ligand engagement, NOD1 and NOD2—the best-characterised NLRs—recruit the protein kinase, RIP2 (also known as RICK), which in turn associates with TRAF2, TRAF5 or TRAF6, enabling TRAF proteins to catalyse their own K63-polyubiquitination as well as that of their target proteins, including RIP2 and IKKγ/NEMO, in conjunction with the Ubc13/Uev1A E2 ligase [35]. Polyubiquitinated RIP2 then recruits the TAK1 kinase complex via TAB2/3-mediated interaction, leading to IKKβ activation by TAK1-catalysed phosphorylation.

Likewise, cytoplasmic RIG-I-like receptors (RLRs), comprising a family of RNA helicases (RLHs), serve as sensors of viral RNA produced during viral replication in infected cells [35]. Upon exposure to their ligands, the RLR receptors, retinoic acid-inducible gene-I (RIG-I) and melanoma differentiation-associated gene 5 (MDA5), associate through CARD domain-mediated interaction with the adaptor protein, mitochondrial antiviral signalling protein (MAVS; also known as IPS-1), triggering the formation of detergent-resistant, prion-like MAVS aggregates [35,50]. Upon polymerisation, MAVS then recruits multiple TRAF proteins, including TRAF2, TRAF3 and TRAF6, which catalyse the K63-linked polyubiquitination of their protein substrates, thereby creating docking sites for UBD-containing proteins [35]. The resulting ubiquitin-mediated protein–protein interactions then enable the assembly of multimeric signalling complexes, leading to the downstream activation of IKK, with the release of NF-κB dimers from IκBs, and the TRAF3-dependent activation of the non-canonical IKK kinases, TANK-binding kinase (TBK1; also known as NAK) and IκB kinase ε (IKKɛ; also known as IKK-i). Both TBK1 and IKKɛ then phosphorylate the IRF-family transcription factors, IRF3 and IRF7, thereby enabling them to cooperate with NF-κB in triggering antiviral immune responses through the induction of type-1 interferons and other antiviral molecules [35,51,52].

#### 2.1.2. NF-κB Activation by TNF-R1

Accumulating evidence reveals an increasingly greater complexity in the ubiquitin-mediated signalling mechanisms that lead to inducible IKK activation in response to a variety of NF-κB-inducing signals, whereby parallel, sequential, alternative or even hybrid types of ubiquitin linkage and ubiquitin-like proteins are involved in signal propagation (reviewed in [21]. An example of this complexity is provided by the NF-κB-inducing pathway emanating from TNF-R1 (Figure 1b). TNF-α recognition causes this receptor to trimerise, triggering the recruitment of the adaptor proteins, TNF receptor-associated death domain protein (TRADD), and the ubiquitin E3 ligases, TRAF2, TRAF5, cellular inhibitor of apoptosis (c-IAP)1 and c-IAP2, which in turn recruit the protein kinase, receptor-interacting protein 1 (RIP1) [35]. Upon ligand-induced signalling complex assembly at the receptor intracellular tail, c-IAP1 and c-IAP2 catalyse the K63-linked polyubiquitination of RIP1 on K377, operating in conjunction with a ubiquitin E2 ligase complex utilising UbcH5. This polyubiquitination event, but interestingly not the RIP1 kinase activity, is then responsible for TNF-α-induced NF-κB activation, which it mediates by enabling the RIP1 polyubiquitin chains to interact with the TAK1 and IKK kinase complexes through the UBDs of TAB2/3 and IKKγ/NEMO, respectively, leading to activation of IKKβ by TAK1-mediated phosphorylation (Figure 1b) [3,21,25,48,53,54,55,56,57,58,59].

Various signalling pathways involving alternative forms of non-degradative ubiquitination and ubiquitin signalling molecules have also been shown to play an important role in NF-κB activation by TNF-R1 (reviewed in [21]). One such ubiquitin-dependent pathway of NF-κB activation involves the linear M1-linked polyubiquitination of RIP1 and IKKγ/NEMO by the linear ubiquitination assembly complex (LUBAC), consisting of HOIP, HOIL-1L and Sharpin, which is recruited to the activated TNF-R1 in a TRADD-, TRAF2- and c-IAP1/2-dependent manner, resulting in IKKβ activation by trans-autophosphorylation [59,60,61,62]. More recently, LUBAC-mediated M1-linked ubiquitination has been shown to also contribute to NF-κB activation by other receptors, including IL-1βR and the TNF-R-family receptors, CD40 and transmembrane activator and CAML interactor (TACI; also known as TNFRSF13B) [21]. Additionally, TNF-α was shown to induce both the K63- and K11-linked polyubiquitination of RIP1, suggesting that distinct TNF-α-induced populations of ubiquitinated RIP1 and, possibly, RIP1 molecules with mixed polyubiquitin chains, contribute to regulate NF-κB signalling [21,41]. A similar situation entailing hybrid K63- and M1-linked polyubiquitin chains has also been reported for IRAK1, IRAK4 and MYD88 in the context of IL-1βR or TLR stimulation [21]. Furthermore, in vitro, IKKγ/NEMO has been shown to be a substrate for K6- and K27-linked polyubiquitination by c-IAP1 and tripartite motif protein 23 (TRIM23), an E3 ubiquitin ligase involved in NF-κB activation by RIG-I-like receptors [21,41]. Indeed, since the N-terminal methionine and any of the seven lysine residues of ubiquitin can form ubiquitin linkages, and both ubiquitination and deubiquitination enzymes (further discussed below) have evolved distinct ubiquitin-linkage specificities, it is conceivable that the precise contribution of the remarkable conformational and functional diversity of polyubiquitin chains to the regulation of NF-κB signalling has just begun to be unravelled.

#### 2.1.3. NF-κB Activation by Antigen Receptors

Upon antigen recognition, in the context of cognate MHC molecules, the TCR initiates a signalling cascade that results in the activation of the serine-threonine kinase, protein kinase C (PKC)θ, within lipid raft microdomains of the immunological synapse [35,63,64]. Activated PKCθ then phosphorylates the molecular scaffold, caspase recruitment domain-containing protein 11 (CARD11; also known as CARMA1), triggering a conformational change, which enables CARD11 to recruit the adaptor protein, BCL10, and the paracaspase, mucosa-associated lymphoid tissue lymphoma translocation protein 1 (MALT1), thereby forming the CARD11-BCL10-MALT1 (CBM) signalling complex (Figure 1c) [35,64]. Genetic evidence demonstrates that each component of the CBM complex is essential for IKKγ/NEMO ubiquitination and IKK activation by TCR stimulation [35,64]. Interestingly, multiple chromosomal and genetic abnormalities affecting each constituent of the CBM complex or anyhow resulting in constitutive CBM complex activation have been reported in various types of lymphoma, including DLBCL and mucosa-associated lymphoid tissue (MALT) lymphoma, where they drive NF-κB activation and oncogenesis [16,17,35,64]. Upon CBM complex formation, BCL10 and MALT1 undergo oligomerisation, thereby enabling MALT1 to bind to TRAF6, causing it to also oligomerise with activation of its E3 ligase activity [35]. MALT1-associated TRAF6 then catalyses its self-polyubiquitination as well as the K63-linked polyubiquitination of its target proteins, including MALT1 and IKKγ/NEMO, via a process that involves the E2 ligase Ubc13/Uev1A (Figure 1c) [35]. BCL10 is also inducibly polyubiquitinated in response to TCR stimulation, although it is unclear whether TRAF6 catalyses this reaction, and both the BCL10 and MALT1 K63-polyubiquitin chains then serve as docking sites that contribute to recruit the IKK complex via the IKKγ/NEMO UBD [35,63].

While TAK1 appears to be recruited to the CBM scaffold via polyubiquitinated TRAF6-mediated interaction with the TAB2/3 UBD, TAK1 activation in response to TCR stimulation appears to also require a further, CBM-independent step (Figure 1c) [22]. Recent studies have suggested a role for the serine-threonine kinase, phosphoinoisitide-dependent kinase (PDK)1, and the molecular adaptor, adhesion- and degranulation-promoting adaptor protein (ADAP), in enabling this additional signalling step by promoting the PKCθ-mediated recruitment of CARD11 and TAK1 to the PKCθ signalosome, thereby resulting in TAK1 activation and IKKβ phosphorylation [22]. There is also evidence of a role for caspase-8, an initiator caspase involved in apoptosis and lymphocyte activation, in positive regulation of NF-κB signalling downstream of the TCR, and both caspase-8 and its regulator, cellular FLICE-like inhibitory protein (c-FLIP), have been found in association with MALT1 in TCR-activated T cells [22]. Notwithstanding, the precise role of caspase-8 in TCR-induced NF-κB activation and the molecular details underlying this role remain to be determined [22,63,65,66]. A similar signalling pathway, utilising many of the same signalling intermediates also involved in TCR-induced NF-κB signalling, including the CBM complex, has been shown to mediate NF-κB activation by B-cell receptor (BCR) stimulation in B lymphocytes (reviewed in [63]).

#### 2.1.4. NF-κB Activation by Genotoxic Stress

Genotoxic stress induced by ionising radiation or chemotherapeutic drugs results in canonical NF-κB activation, which enables cells to survive while the DNA damage is being repaired. This pathway of NF-κB activation involves the trafficking of signalling intermediates between the nucleus and the cytoplasm (reviewed in [35,67]). The NF-κB-inducing signal is initiated in the nucleus by the DNA damage sensors, ataxia telangiectasia mutated (ATM) kinase and poly(ADP-ribose)-polymerase-1 (PARP-1), which, upon binding to DNA strand breaks, synthesizes poly(ADP-ribose) (PAR) chains attached to itself and other target proteins [21,29,35,67,68]. Both enzymes are essential for DNA damage-induced canonical NF-κB signalling [21,29,35,67,68]. Following dissociation from damaged DNA sites, PAR-conjugated PARP-1 proteins recruit IKKγ/NEMO (devoid of any IKK catalytic subunit) to the nucleus to assemble a nuclear signalosome comprising activated ATM, IKKγ/NEMO, and the E3 small ubiquitin-like modifier (SUMO)-protein ligase, protein inhibitor of activated STAT protein y (PIASy) [21,35,67]. PARP-1 signalosome assembly then enables the PIASy-mediated IKKγ/NEMO SUMOylation on K277 and K309 via a process that requires the E2 ligase, Ubc9, the PAR-mediated PIASy modification, and an involvement of the death domain-containing proteins, p53-induced death domain protein (PIDD)1 and RIP1 [35,67]. Importantly, PARP-1 signalosome assembly also enables the ATM-dependent phosphorylation of IKKγ/NEMO on S85 [35,67].

In addition to mediating this essential nuclear function, DNA damage-activated ATM is exported from the nucleus to the cytoplasm, where it binds to TRAF6 via its TRAF-interaction motif. This binding then activates the TRAF6 E3 ubiquitin ligase activity, leading to TRAF6-mediated self-polyubiquitination, in a reaction catalysed in conjunction with the E2 ubiquitin-conjugating enzyme, Ubc13 [35,41,67]. The newly formed TRAF6 polyubiquitin chains then trigger the recruitment of c-IAP1, resulting in the assembly of the ATM-TRAF6-c-IAP1 signalling complex, which in turn recruits the TAK1 and IKK kinase complexes via TAB2- and IKKγ/NEMO-mediated interactions, respectively, resulting in TAK1 activation by trans-autophosphorylation [69]. However, in addition to these cytoplasmic signalling events, efficient IKKβ phosphorylation requires the nuclear export of SUMOylated IKKγ/NEMO, generated in the PARP-1 signalosome [67,70]. Upon integration in the cytoplasmic IKK kinase complex, SUMOylated IKKγ/NEMO is monoubiquitinated on K285 by the c-IAP1 ubiquitin E3 ligase bound to the ATM-TRAF6 signalling complex, leading to TAK1-mediated IKKβ phosphorylation and NF-κB activation [69,71]. Therefore, IKKγ/NEMO monoubiquitination appears to integrate the nuclear signalling pathway driven by the PARP-1 signalosome, with the cytoplasmic ATM-driven pathway induced in response to genotoxic stress [69]. Precisely how IKKγ/NEMO monoubiquitination enables IKKβ phosphorylation is unclear. However, it is possible that it induces a conformational change in the IKK complex that makes IKKβ more accessible to TAK1 [34,35,72,73].

Although canonical NF-κB activation by ionising radiation and chemotherapy is transient, it has nonetheless been shown to contribute to both radio- and chemo-resistance in cancer [74]. This ability of NF-κB to promote tumour-cell survival following treatment with DNA-damaging agents has been extensively demonstrated in cell lines and primary tissues, both in vitro and in vivo, in a wide range of tumour types, including breast carcinoma, squamous cell carcinoma and thyroid carcinoma [75,76,77,78]. Likewise, most, if not all, genotoxic therapeutic agents, including paclitaxel, vinblastine, vincristine, doxorubicin, 5-fluorouracil, cisplatin and tamoxifen, have been shown to induce effective NF-κB activation [75,77]. The products of various NF-κB target genes, most notably *cyclin D1*, *BCL-2*, *Bcl-X_L_*, *survivin* and *XIAP*, have been implicated, each in diverse oncological contexts, as mediators of NF-κB-dependent tumour-cell survival leading to radiotherapy and/or chemotherapy resistance [79,80]. Therefore, the therapeutic inhibition of the NF-κB pathway has also been pursued in order to enhance the clinical effects of radio- and chemo-therapy in cancer (further discussed below).

### 2.2. The Non-Canonical NF-κB Pathway

**Figure 2:**
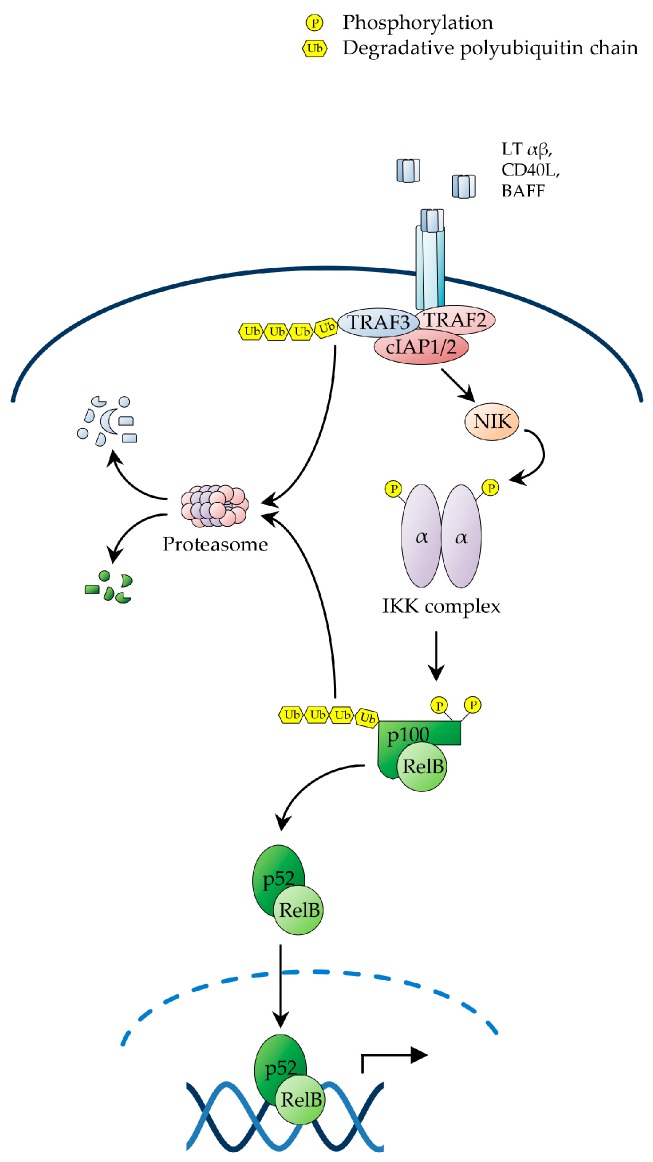
The non-canonical NF-κB pathway. Schematic representation of the non-canonical pathway of NF-κB activation. Activation of this pathway depends on the signal-induced stabilisation of NIK and subsequent NIK-mediated phosphorylation of IKKα on T-loop serine residues. In the absence of receptor stimulation, NIK is constitutively degraded via the ubiquitin-proteasome pathway controlled by the activity of the E3 ubiquitin ligase, c-IAP1/2. Upon ligand-mediated receptor engagement, TRAF3 is degraded with subsequent stabilisation of NIK, which in turn phosphorylates IKKα, leading to C-terminal ubiquitination and proteasome-mediated processing p100. NIK, NF-κB-inducing kinase.

The non-canonical or alternative NF-κB pathway is induced by a distinct group of TNF-family ligands, including lymphotoxin β (LTβ), CD40 ligand (CD40L), B-cell activating factor (BAFF), receptor activator of NF-κB ligand (RANKL), TNF-related weak inducer of apoptosis (TWEAK) and tumor necrosis factor superfamily member 14 (TNFSF14; also known as LIGHT) [4,45], and governs developmental and immune processes, such as secondary lymphoid organogenesis, B-cell survival and maturation, bone morphogenesis and dendritic-cell activation [81,82,83,84,85,86,87,88,89]. In contrast to canonical NF-κB signalling, which is subject to rapid and transient activation, the non-canonical NF-κB pathway is activated with distinctively slower kinetics. Additionally, it does not require IKKβ or IKKγ/NEMO, nor does it involve the proteolysis of canonical IκBs. Instead, it exclusively relies on IKKα and the signal-induced processing of p100, which releases RelB/p52 heterodimers (Figure 2) [4,21,25,34].

Receptor engagement by ligands activating non-canonical NF-κB signalling triggers a signalling cascade that results in the stabilisation of NF-κB-inducing kinase (NIK), which in turn, upon accumulation in cells, phosphorylates IKKα on T-loop serine residues, S176 and S180, leading to IKKα activation [4,34]. Activated IKKα then phosphorylates p100 on C-terminal serine residues, S866 and S870, thereby creating a docking site for SCF^βTrCP^, similar to the destruction motif generated by signal-induced phosphorylation of IκBs [25,34]. Upon recruitment to p100, SCF^βTrCP^ catalyses the K48-linked polyubiquitination of p100 on lysine residue, K856, in conjunction with a Ubc4/5 ubiquitin E2 ligase, thereby targeting the C-terminal, IκB-like domain of the precursor for partial proteolysis by the proteasome [25,34]. A proteasomal stop signal, comprising a glycine rich region (GRR) present between the RHD and ARD domains of p100, protects the p100 N-terminal domain from proteolysis, thereby enabling the generation of the mature p52 subunit, which together with RelB forms RelB/p52 heterodimers, which translocate to the nucleus to regulate the transcriptional programme governed by non-canonical NF-κB signalling (Figure 2) [4,45,90].

In unstimulated cells, NIK is constitutively bound to a complex comprising TRAF2, TRAF3 and c-IAP1 or c-IAP2, whereby TRAF3 serves as an adaptor interacting directly with both NIK and TRAF2, which in turn binds to c-IAP1/2, enabling it to catalyse the NIK K48-linked polyubiquitination, resulting in constitutive NIK degradation by the proteasome [4,21,25,27]. Upon receptor engagement, the NIK-associated TRAF2/TRAF3/c-IAP1/2 complex is recruited to the receptor via TRAF3-mediated interaction, enabling TRAF2 to catalyse the non-degradative K63-polyubiquitination of c-IAP1/2. This redirects the c-IAP1/2 E3 ligase activity from NIK to TRAF3, causing the c-IAP1/2-mediated K48-polyubiquitination of TRAF3 [4,21,25,27]. The resulting proteosomal degradation of TRAF3 then destabilizes the TRAF2/TRAF3/c-IAP1/2 complex, leading to NIK dissociation from c-IAP1/2, with consequent NIK stabilization and accumulation of newly synthesized NIK [4,21,25,27,91]. As the NIK kinase domain adopts an intrinsically active conformation, accumulated NIK does not require any additional phosphorylation event for activation, and consequently binds to and phosphorylates IKKα, leading to p100 processing and NF-κB activation (Figure 2) [4,21,25,27]. Interestingly, a number of genetic alterations promoting NIK stabilization and p100 processing, such as *c-IAP1/2* and *TRAF3* deletions and *NIK* amplifications, have been reported in multiple myeloma, where they drive constitutive NF-κB activation and multiple myeloma cell survival [21,24,26,28,34,68,92].

In addition to their roles as NF-κB precursors, both p100 and p105 can serve as IκB inhibitory proteins by means of their C-terminal IκB-like ARD domains [4,8,25,33]. Degradative ubiquitination of the precursors may therefore serve a dual purpose: On the one hand, it may promote their partial proteolysis to produce active NF-κB subunits, which can form homodimers or heterodimeric complexes with other NF-κB-family proteins; on the other hand, it may result in their complete degradation, thereby liberating sequestered NF-κB dimers from their interaction with inhibitory C-terminal IκB-like domains [4,8,25,33]. Notably, in contrast to the signal-induced processing of p100, which is tightly regulated by the non-canonical NF-κB pathway via IKKα-mediated p100 phosphorylation, with minimal basal processing in the absence of stimulation, the processing of p105 to p50 is largely a constitutive process that may occur either co- or post-translationally [25]. Notwithstanding, in addition to being subject to signal-induced partial proteolysis, controlled by SCF^βTrCP^-dependent ubiquitination, p100 is constitutively targeted for complete degradation, which facilitates efficient, stimulus-induced non-canonical NF-κB activation, via a process that depends on the glycogen synthase kinase 3 (GSK3)-mediated phosphorylation of p100 at C-terminal serine residue, S707, and subsequent ubiquitination catalysed by an SCF complex containing the F-box protein, FBXW7α [24,25,33].

### 2.3. Termination of NF-κB Signalling

The feedback control and timely termination of the NF-κB response are essential to ensure the restoration of homeostasis and prevent excessive inflammation, tissue damage and the onset of neoplasias. To maintain transience and ensure the prompt cessation of NF-κB signalling, multiple negative feedback mechanisms have evolved to control the NF-κB pathway at various levels. The first discovered and best characterised attenuation mechanism consists in the resynthesis of IκB proteins following NF-κB activation [4,25,27,28]. All *IκB* genes, including those encoding canonical IκBs and NF-κB precursors, contain κB sites in their promoters [72]. An essential negative feedback mechanism is mediated by IκBα, which upon its resynthesis, helps to terminate the NF-κB response by virtue of its ability to enter the nucleus, dissociate NF-κB complexes from DNA, and export them into the cytoplasm [25,28,72].

A number of additional downstream inhibitory mechanisms are mediated by various types of post-translational modification of NF-κB subunits, including site-specific phosphorylation, acetylation and ubiquitination, which attenuate the NF-κB response by directly affecting the protein stability, DNA-binding affinity and/or transcriptional activity of nuclear NF-κB dimers and/or their interactions with transcriptional cofactors [25,27,28]. For instance, in LPS-stimulated macrophages, nuclear IKKα has been shown to phosphorylate the C-terminal domains of RelA and c-Rel within DNA-bound NF-κB complexes, thereby accelerating NF-κB protein turnover with consequent downregulation of inflammatory gene expression [93]. Nuclear RelA has also been reported to undergo proteosomal degradation, which contributes to the termination of NF-κB-dependent transcription, through a mechanism mediated by the ubiquitin E3 ligases, suppressor of cytokine signalling-1 (SOCS-1) and PDZ and LIM domain protein 2 (PDLIM2) [25,27,28]. Several other mechanisms participate in the cessation of NF-κB signalling, including RelA acetylation, which diminishes the DNA-binding affinity of RelA-containing complexes and affect their interaction with both histone acetyltransferases (HATs) and histone deacetylases (HDACs) [28]. Likewise, site-specific RelA dephosphorylation by wild-type p53-induced phosphatase 1 (WIP1) has been shown to weaken the RelA interaction with the transcriptional coactivator, p300, resulting in an attenuation of RelA-dependent gene transcription [29]. Moreover, the oxidation of redox-sensitive cysteine residues in the DNA-binding domains of NF-κB subunits has been suggested to dampen the NF-κB response [28,34].

In addition to these downstream processes, multiple feedback mechanisms regulate NF-κB signalling components operating either at the level or upstream of the IKK complex. One such mechanism relies on the intrinsic self-limiting capacity of the IKK complex, dependent upon the IKKβ-mediated phosphorylation of IKKγ/NEMO on serine residue, S68, and the IKKβ auto-phosphorylation on C-terminal serine residues, resulting in a disruption of essential structural motifs and interactions of IKK subunits [21]. IKKβ further catalyses the BCL10 phosphorylation, which has been shown to downregulate TCR-induced NF-κB activation [21]. Additionally, a number of phosphatases, including protein phosphatase (PP)2A and PP2C, contribute to inhibit NF-κB activation by dephosphorylating T-loop serines in the catalytic IKK subunits [21,23].

Notably, accumulating evidence demonstrates the importance of negative feedback mechanisms affecting the ubiquin system. Recent studies have shown that the NEMO-like adaptor, optineurin, plays an important role in the negative regulation of TNF-α-induced NF-κB activation by competing with NEMO for binding to the polyubiquitin chains of several IKKγ/NEMO-interacting proteins [21,27]. Additionally, several deubiquitination enzymes (DUBs), including A20 (also known as tumor necrosis factor α-induced protein 3; TNFAIP3) and the tumour suppressor, CYLD (also known as cylindromatosis), implicated in familial cylindromas, have been shown to control critical signalling steps upstream of the IKK complex [4,5,35]. Both A20 and CYLD are direct transcriptional targets of NF-κB and, as such, are rapidly induced upon NF-κB activation, thereby providing essential feedback mechanisms promoting the cessation of the NF-κB response. A20 and CYLD both mediate this function, at least in part, by hydrolysing the K63-polyubiquitin chains of a number of signalling molecules involved in IKK activation, including RIP1/2, TRAF proteins, NOD2, MALT1 and IKKγ/NEMO itself [4,21,35,65]. Interestingly, A20 may further limit NF-κB signalling by virtue of its ubiquitin-editing function, whereby upon removing the RIP1 K63-polyubiquitin chains via the DUB activity mediated by in its N-terminal domain, it can catalyse the RIP1 K48-linked polyubiquitination via the ubiquitin E3 ligase activity in its C-terminal domain, thereby targeting RIP1 for proteosomal degradation [5,35]. Several other DUBs have been implicated as negative regulators of canonical NF-κB signalling. These include OTU domain-containing protein 7B (OTUD7B; also known as cezanne), ubiquitin-specific peptidase 11 (USP11), USP15 and USP21, each exhibiting distinct ubiquitin-linkage specificity [41]. Interestingly, some of the more recently identified DUBs antagonising NF-κB activation have been shown to target M1-type ubiquitin linkages. For instance, otulin (also known FAM105B) has been reported to HOIP-mediated interaction and specifically remove M1 linear polyubiquitin chains [5,21]. Moreover, in addition to cleaving K63 ubiquitin linkages, CYLD has been shown to degrade M1-linked polyubiquitin chains from various components of the TNF-R1 and NOD2 signalling complexes, including RIP1. Curiously, A20 also has the ability to bind to M1 polyubiquitin chains, but appears to have the opposite effect on ubiquitin chain stability, by preventing their removal [41].

An additional mechanism of NF-κB inhibition that is being viewed with increasing interest is mediated by the tumour suppressor, WW domain-containing oxidoreductase (*WWOX*), which is frequently inactivated by gene mutation, deletion or chromosomal translocation in multiple types of haematological and solid cancer [94,95,96,97,98,99,100,101,102]. WWOX has been shown to negatively regulate canonical NF-κB signalling by means of its ability to directly bind to IκBα and, thereby, impede proteasome-mediated IκBα proteolysis [94,97,98]. This inhibitory activity of WWOX has additionally been implicated in the suppression of canonical NF-κB activation by the HTLV-1 protein, Tax, in adult T-cell leukaemia (ATL) [94]. Therefore, genetic WWOX inactivation in cancer contributes to constitutive NF-κB activation. Interestingly, in addition to the aforementioned genetic mechanisms of inhibition, *WWOX* is negatively regulated by non-canonical NF-κB signalling and, therefore, appears to mediate a mechanism of cross-amplification of canonical NF-κB activity by the non-canonical NF-κB pathway [94].

Far fewer negative feedback mechanisms have been reported for the non-canonical NF-κB pathway. An important checkpoint in this respect is mediated by TRAF3. In addition to being transcriptionally upregulated by NF-κB to promote NIK degradation, leading to p100 stabilization and attenuation of non-canonical NF-κB signalling, TRAF3 is subject to post-translational stabilisation by means of OTUD7B, which targets TRAF3 for deubiquitination of its K48-linked polyubiquitin chains, thereby preventing NIK accumulation and the processing of p100 [21,103]. IKKα-mediated NIK phosphorylation has also been reported to accelerate NIK turnover, thereby contributing to the feedback regulation of non-canonical NF-κB signalling [41]. Additionally, microRNA-146a may negatively regulate both canonical and non-canonical NF-κB activation by downmodulating the expression of IRAK1, TRAF6 and RelB [104].

## 3. Therapeutic Targeting of the NF-κB Pathway in Cancer

The central role that NF-κB plays in a vast range of human malignant and non-malignant pathologies has catalysed an intensive effort by the pharmaceutical industry and academic laboratories over the past two and a half decades to develop a specific NF-κB inhibitor for clinical indication in these diseases [2,3,11,105]. In 2006, a survey had already counted no fewer than 750 candidate therapeutics designed to target the NF-κB pathway, and this number is certain to have grown considerably over the past decade [3]. These compounds comprise a disparate variety of chemical classes, including peptidomimetics, small molecules, small interfering RNAs and microbial products and their derivatives. Many of them function as general inhibitors of NF-κB signalling, while for many others the mode of action is poorly understood [3,21,105]. Yet, notwithstanding the tremendous progress made in recent years towards unravelling the intricate signalling networks governing the NF-κB pathway and its functions, and the intensive efforts and mighty investments committed to develop a specific, clinically useful NF-κB inhibitor, the output of pharmacological interventions has been disappointingly scant, with a continuing dismaying absence of a specific NF-κB or IKK inhibitor in the clinical anticancer armamentarium. The insurmountable challenge, having precluded the clinical success of these NF-κB-targeting approaches, has been to achieve contextual selective inhibition of the NF-κB pathogenetic activity, while preserving the essential physiological functions of NF-κB. By contrast, the efforts so far have resulted in drug candidates often causing the global suppression of NF-κB and its pleiotropic and ubiquitous functions, leading to severe dose-limiting toxicities.

**Figure 3:**
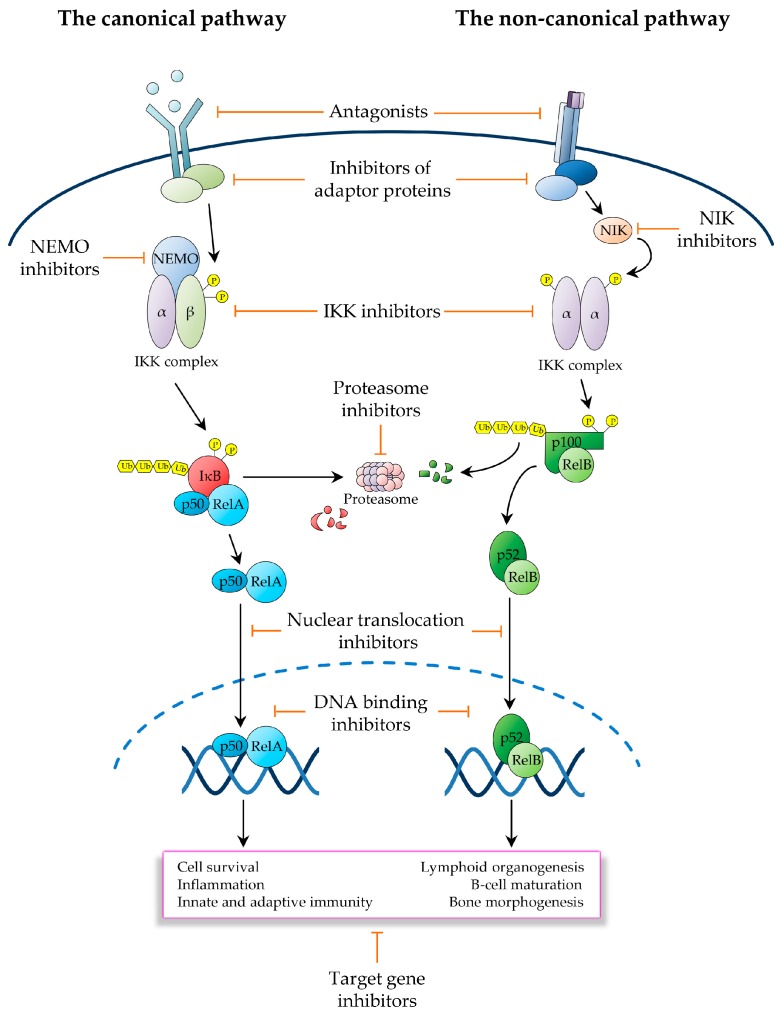
Schematic representation of the main strategies utilised to therapeutically inhibit the NF-κB signalling pathway. Depicted are the canonical (**left**) and non-canonical (**right**) pathways of NF-κB activation. Also depicted are the main therapeutic strategies utilised to inhibit these NF-κB signalling pathways in the oncological context.

This review examines the main approaches utilised to therapeutically target NF-κB, with a focus on oncology, illustrating for each approach the underlying rationale and most representative classes of compounds, while emphasising emerging strategies and some of the most promising future directions. For ease of discussion, we have broadly classified NF-κB-targeting therapeutics on the basis of their mode of action, depending upon the level of the NF-κB signalling pathway at which they operate (Figure 3). Given the breadth of the basic and translational research in this area, it has not been possible to cover all relevant aspects of the preclinical and clinical pharmacology of the vast number of molecules generated to inhibit NF-κB. For further information on these molecules and their effects, we therefore refer to the excellent reviews that have previously covered these topics [2,3,105,106,107].

### 3.1. Inhibitors Operating Upstream of the IKK Complex

NF-κB can be activated by a multitude of stimuli, which initiate distinct signalling pathways that converge on the IKK complex (Figure 1 and Figure 2). From a clinical standpoint, this paradigm provides a significant opportunity for therapeutic interventions aimed at interfering with pathogenic NF-κB activation (Figure 3). An important area of drug discovery in this context has been the TNF-R superfamily, which governs diverse physiological processes, including inflammation, cell survival and lymphoid organogenesis, and drives the pathogenesis of chronic inflammatory diseases as well as multiple cancer types [108,109]. TNF-Rs comprise a family of 29 structurally-related receptors, which are bound by 19 ligands of the TNF superfamily [108]. Due to the important roles that TNF-Rs play in widespread human pathologies, there has been a great deal of interest over the past few decades in developing therapeutics targeting these receptors or their ligands [110]. The first such a therapeutic has been infliximab, a TNF-α-specific neutralising antibody, which was approved in 1998 for the treatment of Crohn’s disease, followed by the approval in the same year of etanercept [108]. Both drugs are currently in clinical use for the treatment of rheumatoid arthritis, psoriasis, psoriatic arthritis, and other forms of chronic arthritis, and infliximab is also in clinical use for Chron’s disease and ulcerative colitis [108]. However, patients treated with these drugs often experience significant side effects, including fevers, chills, nausea, shortness of breath, tachycardia and hypotension. Indeed, the dose-limiting toxicities and immunosuppressive activities of TNF-R signalling inhibitors have precluded the broader clinical development of these agents beyond chronic inflammatory diseases, in particular in the area of oncology [109]. Notwithstanding, a few molecules interfering with TNF-R signalling have found indication in niche areas of oncology. For instance, the human TNF-α analogues, tasonermin (Beromun), has been clinically approved as an adjunct therapy to surgery for sarcoma to prevent or delay amputation and to treat unresectable soft-tissue sarcoma of the limbs [111]. Likewise, brentuximab (Vedotin, Adcetris), a toxin-conjugated chimeric antibody targeting the TNF-R-family receptor CD30 (also known as TNFRSF8), is approved for the treatment of Hodgkin’s lymphoma and anaplastic large-cell lymphoma (ALCL) (Table 1), two cancer types that express particularly high surface levels of CD30 [112].

In B-cell lymphoma and leukaemia, the NF-κB pathway can be inhibited by therapeutic agents targeting proximal signalling events downstream of the BCR. Upon antigen engagement, the BCR triggers a signalling cascade that involves receptor-associated CD79A/B heterodimers, SRC-family protein tyrosine kinases and Burton tyrosine kinase (BTK), which, as in the case of TCR-induced signalling, leads to the downstream the assembly of a PKC signalosome (mediated by PKCβ, rather than PKCθ, as in T cells) and the CBM signalling complex (Figure 1c) [65]. BTK is an essential signalling intermediate in the BCR-induced pathway leading to NF-κB activation and B-cell survival [113]. Notably, while BTK is expressed in virtually all cells of the haematopoietic lineage, except for T cells and plasma cells, its functions in NF-κB activation and cell survival appear to be dispensable outside of B-cell lineage [114]. Consequently, BTK has been developed into an effective therapeutic target upstream of IKK in various types of B-cell malignancy, including chronic lymphocytic leukaemia (CLL), mantle-cell lymphoma (MCL), follicular lymphoma (FL), DLBCL and acute lymphoblastic leukaemia (ALL) [113]. The first-in-class oral BTK inhibitor, ibrutinib (PCI-32765), has demonstrated impressive clinical responses in clinical trials as a single agent and has been subsequently approved by the FDA for the treatment of refractory MCL (November 2013), CLL (February 2014) and Waldenström’s macroglobulinemia (WM; January 2015) [115]. Ibrutinib irreversibly binds to cysteine residue, C481, in the active site of BTK, thereby inhibiting BTK phosphorylation on T223 and resulting in loss of BTK function, NF-κB inhibition and induction of tumour-cell apoptosis [114]. Owing to BTK’s restricted pattern of expression, ibrutinib is generally relatively well tolerated, causing for the most part only transient adverse effects, such as diarrhoea, nausea, vomiting, hypertension, urinary and upper respiratory tract infections, fatigue, arthralgia, pyrexia, and peripheral oedema [116]. However, secondary resistance is almost inevitable, and tumours with oncogenic NF-κB-pathway alterations affecting signalling events downstream of BTK, such *CARD11* mutations and *A20* mutations and deletions, are naturally refractory to this agent, thereby limiting its clinical utility, especially in patients with aggressive NF-κB-pathway mutated lymphomas (Table 1) [113].

The gene encoding the adaptor protein, MYD88, which mediates NF-κB and MAPK activation downstream of all TLRs, with the exception of TLR3 [117], is recurrently mutated in haematological malignancies, such as DLBCL, WM and CLL, where it induces constitutive NF-κB and STAT3 activation, thereby promoting cancer-cell survival and oncogenesis [117,118]. These findings provide a strong rationale for therapeutically targeting TLR signalling in certain types of lymphoma and leukaemia. Congruently, preclinical studies have demonstrated that the antisense oligonucleotide TLR inhibitor, IMO-8400, which specifically targets TLR7, TLR8 and TLR9, is effective in diminishing the growth of WM and DLBCL xenografts, driven by gain-of-function MYD88 mutations [118]. A phase I/II trial of IMO-8400 is ongoing in patients in WM and DLBCL (Table 1), and second generation TLR 7/TLR 8/TLR9 inhibitors are currently in development. Another strategy aimed at therapeutically targeting TLR signalling in cancer is directed at the downstream protein kinases, IRAK1/4. This strategy has demonstrated preclinical efficacy in tumours harbouring MYD88 mutations, and preclinical studies of IRAK1/4 small-molecule inhibitors have reported encouraging preliminary results in melanoma [119], myelodysplastic syndrome (MDS) [120] and T-cell acute lymphoblastic leukaemia (T-ALL) [121,122].

The non-canonical NF-κB pathway plays an important role in the pathogenesis of multiple myeloma, where it is constitutively activated by recurrent genetic alterations, including *NIK* amplifications and *c-IAP1/2* and *TRAF3* deletions [10,12,14]. Recent studies suggest an additional role for non-canonical NF-κB signalling in other types of malignancy, such as DLBCL [45]. Within this signalling pathway, NIK is an especially attractive target, owing to its central role in controlling IKKα phosphorylation and p100 processing (Figure 3). Two NIK small-molecule inhibitors, which were recently developed by Amgen, AM-0216 and AM-0561 [123], have demonstrated significant therapeutic activity in multiple myeloma cells, in vitro [124,125,126]. However, further studies are required to evaluate their potential therapeutic efficacy, in vivo.

c-IAP proteins play an important role in tipping the balance between canonical NF-κB activation and inhibition of non-canonical signalling. Upon TNF-R1 stimulation, receptor-associated c-IAP proteins contribute to recruit the LUBAC complex by ubiquitinating various signalling intermediates, resulting in NF-κB activation [127]. By contrast, in the non-canonical pathway, c-IAP-mediated ubiquitination reactions result in constitutive NIK degradation, thereby dampening non-canonical NF-κB activation [45]. Underscoring these opposing functions of c-IAPs in canonical and non-canonical NF-κB activation, *c-IAP* genes are subject to both amplification and deletion in human cancer [127]. Consequently, the aim of the therapeutic interventions targeting c-IAP proteins in cancer is to inhibit canonical NF-κB activation, without interfering with non-canonical NF-κB signalling. The endogenous c-IAP antagonist, second mitochondria-derived activator of caspases (SMAC), provides an attractive target for achieving this aim. In response to apoptosis-inducing stimuli, SMAC is released from mitochondria into the cytoplasm, where it binds to the conserved c-IAP domain, baculovirus IAP repeat (BIR), via its N-terminal AVPI tetrapeptide, thereby neutralising the prosurvival activity of c-IAPs [128]. This paradigm has served as a reference point for the development of drug mimetics that act as selective c-IAP inhibitors. At least two such compounds, i.e., LCL161 and birinapant (TL32711), have now entered clinical trials in patients with solid cancers, such as ovarian serous carcinoma (Table 1) [129]. Although these compounds have been shown to promote cancer-cell death by stimulating an increase in cytokine production, they have also been reported to have severe dose-limiting toxicities, in particular the onset of cytokine-release syndrome, which significantly restrict their clinical application [130]. Other common, but less severe, side effects include vomiting, nausea, fatigue, and anorexia [131].

Therefore, although targeting upstream NF-κB signalling components has yielded tangible clinical results in the treatment of certain cancers and represents an attractive therapeutic strategy from the standpoint of achieving a degree of tissue- and context-specificity, this approach has so far been limited by the onset of dose-limiting adverse effects, inherent cancer recalcitrance and/or an early onset of secondary drug resistance [109,113,130]. Nevertheless, as the clinical efforts in this area are relatively new and the underlying research is constantly advancing the understanding the intricate upstream signalling networks leading to IKK activation, targeting these networks as a means of therapeutic intervention holds promise for the future development of safe and effective anticancer therapeutics. While strictly speaking, most therapies interfering with upstream signalling intermediates will lack NF-κB-selective specificity, as they will inevitably also affect pathways beyond the NF-κB pathway, this limitation may be overcome at least in part by targeting protein-protein interactions involved in IKK activation, an area that remains largely unexplored and is likely to deliver effective and more specific NF-κB-targeting therapeutics. Although developing molecules that affect protein–protein interactions presents clear challenges, the heavy reliance of NF-κB activation pathways upon adaptor molecules and inducible formation of multimeric signalling complexes, coupled with the flourishing progress being made in unravelling new interactions and their regulation, is certain to catalyse the translational research efforts to develop NF-κB inhibitors in the future.

### 3.2. IKK Inhibitors

Owing to its central role as the signal integration hub for NF-κB activation pathways (Figure 1 and Figure 2), the IKK complex has been the focus of significant drug discovery efforts since its discovery in 1996. However, while a vast spectrum of inhibitors has been developed throughout the years, only a few of these agents have ever been entered into clinical trials, and none has been clinically approved (Figure 3). A seminal paper by Michael Karin and colleagues in 2007 irreversibly tempered the initial enthusiasm over these agents as candidate NF-κB-targeting therapeutics [132]. This paper demonstrated that pharmacological IKKβ inhibition results in elevated systemic levels of IL-1β, owing to increased pro-IL-1β processing and IL-1β secretion by macrophages and neutrophils upon bacterial infection or exposure to endotoxin, leading to overt systemic inflammation and lethality in mice [132]. These severe on-target toxicities of IKKβ inhibitors have exposed the serious consequences of long-term IKK inhibition, which combined with the associated immunodeficiency and increased risks of malignancies arising from the liver, skin and other tissues [9,11,107,132,133,134], have irrevocably undermined any research efforts to clinically develop IKKβ-targeting therapeutics.

Despite their amino acidic sequence similarity, IKKα and IKKβ play largely distinct roles in NF-κB activation [107,135], whereby canonical NF-κB signalling strictly relies upon IKKβ-mediated phosphorylation of IκBs, while non-canonical NF-κB activation exclusively relies on IKKα. Nonetheless, IKKα also contributes to canonical NF-κB-dependent transcriptional responses by modulating the nuclear activities of RelA, histones and various transcriptional co-activators and co-repressors [2,136]. While the focus of the translational research has been on developing specific IKKβ inhibitors, these molecules often also target IKKα [3,136], owing to the high degree of similarity between these kinases.

IKKα/IKKβ inhibitors can be broadly classified into three major groups on the basis of their mode of action: ATP analogues, allosteric modulators, and agents interfering with the kinase activation loops [3,136]. ATP analogues are the largest group and comprise both natural products such as β-carboline, and small-molecule inhibitors such as SPC-839 (Celgene). SPC-839 is a synthetic quinazoline analogue, which exhibits an approximately 200-fold selectivity for IKKβ over IKKα [2,3,105,137]. The imidazoquinoxaline derivative, BMS-345541, binds to an allosteric pocket present on both IKKα and IKKβ and is an example of an allosteric modulator, having a 10-fold higher selectivity for IKKβ than IKKα. Curiously, while BMS-345541 does not interfere with ATP binding to IKKβ, it disrupts ATP binding to IKKα [2,3,11,105,137,138]. BMS-345541 has so far mainly been tested in vitro, and demonstrated some efficacy against collagen-induced arthritis in mouse models [2,3,11,105,137,138,139,140,141]. Tiol-reactive compounds, including parthenolide, arsenite and epoxyquinoids, inhibit IKKβ by interacting with T-loop cysteine residue, C179 [3,105]. Although their exact mode of action is not well understood, this class of compounds appears to induce post-translational modifications that curtail IKKβ activity [3,105]. Parthenolide displays poor bioavailability and therefore has limited therapeutic application [142]. A new IKKβ inhibitor more recently developed by Sanofi-Aventis, SAR-113945, has been investigated in four different clinical trials in non-oncological patients, with initial promising results [143]. However, SAR-113945 has failed to demonstrate clinical efficacy in follow-on clinical trials, underscoring the challenge of striking an acceptable balance between efficacy and adverse side-effects [143]. Overall, the disappointing clinical performance of IKKβ inhibitors has considerably dampened the interest in this class of agents, and this trend is likely to continue in the future, as shown by the dramatic decline in the number of patent applications filed on these agents in recent years [107].

A significant effort has also been made towards developing molecules that target the IKKγ/NEMO scaffold (Figure 3), using one of three main targeting strategies aimed at perturbing either the IKKγ/NEMO interaction with catalytic IKK subunits, IKKγ/NEMO dimerization, or IKKγ/NEMO ubiquitination. The IKKγ/NEMO interactions with IKKα and IKKβ involve the kinase C-terminal NEMO-binding domain (NBD), which consists of the hexapeptide amino acid sequence, LDWSWL [135,136]. Peptidomimetics of this sequence have been shown to disrupt the IKKγ/NEMO–IKKα/IKKβ interaction and accordingly inhibit NF-κB activation by various upstream signals. However, the therapeutic utility of these agents is limited by their poor bioavailability and significant instability, in vivo [135]. Notwithstanding, small-molecule inhibitors of the IKKγ/NEMO–IKKα/IKKβ interaction may have the potential to provide useful agents to pharmacologically target the NF-κB pathway [136]. Following publication of the crystal structure of the IKKγ/NEMO–IKKβ interface, four phenothiazine derivatives were developed and demonstrated to have inhibitory effects on NF-κB activation in macrophages, in vitro [135]. However, further studies are required to determine whether these agents retain their inhibitory activity, in vivo, and crucially whether they are suitable for further development, especially in consideration of the safety concerns raised by IKKβ inhibitors [135]. Peptidomimetics were also developed to interfere with IKKγ/NEMO dimerisation [144]. However, to our knowledge, no significant preclinical development has ever been reported on any of these agents. Moreover, there has been significant interest in generating agents targeting the IKKγ/NEMO UBAN (i.e., UBD in the ABIN proteins and NEMO) domain, which transduces IKK activation signals by mediating the inducible interactions between IKKγ/NEMO and the polyubiquitin scaffolds of other signalling components [21,145]. Several agents have been shown to disrupt these ubiquitin-dependent IKKγ/NEMO interactions, including peptidomimetics [57], as well as small molecules such as anthraquinone derivatives of emodin [145]. However, despite the ability of some IKKγ/NEMO-targeting agents to modulate NF-κB activation without interfering with the IL-1β release, there appear to have been no clinical trials ever initiated to investigate the clinical safety and efficacy of these molecules.

The IKK-related kinase, TBK1 was originally discovered due to its involvement in NF-κB activation by PKCε-mediated signals [146]. As well as IKKε, TBK1 primarily regulates the activation of IRF-family factors, such as IRF3, IRF5 and IRF7, by RLRs and other receptors [147,148,149], but also phosphorylates RelA to enhance its transcriptional activity [136,146,150]. TBK1 and IKKε share a 64% sequence identity, but display only 27% identity with classical IKKs, and are both involved in inflammatory responses, oncogenesis, and insulin resistance [107]. Moreover, IKKε has been found overexpressed in breast and ovarian carcinoma, while TBK1 cooperates with RAS in promoting malignant transformation, in vitro, and is overexpressed in the carcinoma of the lung, colon and breast [151,152,153,154,155,156]. The antiinflammatory agents, BX765 and CYT387 (momelotinib), were originally developed as inhibitors of 3-phosphoinositide-dependent protein kinase 1 (PDK1) [157] and Janus kinases (JAKs), respectively, but were subsequently shown to also have potent inhibitory activity against TBK1 and IKKε [158]. Consequently, BX765 and CYT387 have been used as starting points for the molecular design of new TBK1 and IKKε antagonists. However, similar to these prototypes, the resulting compounds also demonstrated broad kinase target specificity [159]. Domainex and Myrexis have since generated more selective TBK1 and IKKε inhibitors, such as DMXD-011 and MPI-0485520, respectively [159]. DMXD-011 displays good drug-like properties, is orally bioavailable and has shown promising therapeutic activity in in vivo inflammatory disease models [160]. Likewise, MPI-0485520 displays good oral bioavailability and has been investigated in mouse models of systemic lupus erythematous, rheumatoid arthritis and other autoimmune disorders, with significant therapeutic activity demonstrated, especially in the context of especially in the context of rheumatoid arthritis [161]. However, further investigations are required to determine the potential clinical benefit resulting from these agents and whether their preclinical efficacy translates to the oncological context [159].

### 3.3. Ubiquitin and Proteasome Pathway Inhibitors

The ubiquitin pathway provides a highly versatile and tightly regulated system of signal propagation and protein degradation conserved throughout the eukarya dominion [162,163,164]. Over the past decade, this system has witnessed a burgeoning interest, not only because of its central importance in signal transduction and protein degradation, but also due to its potential for serving as a treasure trove of drug targets for therapeutic intervention in a wide range of malignant and inflammatory pathologies [162,165]. The ubiquitin pathway consists of a three-step enzymatic process, catalysed by as many protein complexes, whereby a ubiquitin-activating enzyme (E1) first binds to and activates a ubiquitin molecule in an ATP-dependent reaction, followed by the sequential transfer of the activated ubiquitin molecule to the active site of a ubiquitin-conjugating enzyme (E2) and, finally, to the target protein through the site-specific recognition and correct positioning by a ubiquitin-protein ligase (E3), which operates in conjunction with the E2 ubiquitin-conjugating enzyme to catalyse the covalent attachment of an 8-kDa ubiquitin molecule to the target site(s) of the protein substrate [25,33,162,165,166,167]. In addition to catalysing the attachment of the first ubiquitin molecule onto the protein substrate, the same three-step enzymatic process conjugates further ubiquitin molecules to form polyubiquitin chains [167].

Ubiquitin-mediated signalling pathways play a central role in the regulation of both canonical and non-canonical NF-κB activation (Figure 1 and Figure 2) [21,24]. They also regulate oncogenesis by participating in either tumour suppression or tumour promotion [21,24,35,162]. Therefore, interfering with the ubiquitin system can affect cancer development and progression in many different ways (Figure 3). Notwithstanding, tumour cells that depend on constitutive NF-κB signalling for survival often display sensitivity to inhibitors of the ubiquitin-proteasome pathway (UPP), owing to the essential role of this pathway in the proteolytic degradation of IκB proteins [6,25,162]. The active proteasome consists of a large 2.4-MDa protein complex, which comprises a 20S catalytic core of a cylindrical shape and two regulatory 19S components forming a lid-like structure at both extremities of the 20S cylinder, and catalyses the proteolysis of substrate proteins via an ATP-dependent process [167].

The first proteasome inhibitors developed were molecules of the class of peptide aldehydes and were subsequently extensively used for preclinical research [168]. The best characterised of these molecules is MG132, which also served as a prototype for the generation of the next classes of proteasome inhibitors that later found common use in the clinical practice [169]. The first proteasome inhibitor ever tested in humans was bortezomib (velcade; formerly known as PS-341), a reversible boronic-acid inhibitor of the 20S catalytic subunit [137,162]. Bortezomib received the approval of the FDA in 2003 for the treatment of refractory multiple myeloma, and is currently in clinical use as a front-line therapy in combination with other agents for the treatment of multiple myeloma and mantle cell lymphoma (MCL) [162]. A number of clinical trials are ongoing to assess the efficacy of bortezomib in further oncological indications, including solid cancers (Table 1). An especially promising area of development for bortezomib and other proteasome inhibitors is their use as part of combination therapies aimed at overcoming radio- and chemo-resistance in cancer. Indeed, bortezomib has been shown to have a strong synergistic activity when used in combination with radiotherapy and/or chemotherapy in various types of haematological and solid cancer [170,171,172,173,174,175]. The second-generation proteasome inhibitor, carfilzomib (Kyprolis), was approved in 2012 as a single agent and in 2016 in combination with dexamethasone, with or without lenalidomide, for the treatment of patients with relapsed or refractory multiple myeloma who have received at least one line of prior therapy. Carfilzomib is an epoxyketone compound, acting as an irreversible proteasome inhibitor to afford prolonged therapeutic inhibition [162], and is currently being investigated in multiple myeloma in combination with other agents, as well as other oncological indications (Table 1). Since both bortezomib and carfilzomib can be only administered intravenously or subcutaneously, the boronic proteasome inhibitor, ixazomib (MLN-9708), was recently developed as an oral therapy and was approved by the FDA in 2015 in combination with lenalidomide and dexamethasone for the treatment of patients with multiple myeloma who have received at least one line of prior therapy [176]. Ixazomib is currently being evaluated in patients with other cancer types and as part of other combination regimens (Table 1).

The human genome encodes two E1 ubiquitin-activating enzymes, UBA1 and UBA5, an estimated fifty E2 ubiquitin-conjugating (UBC) enzymes, and more than six hundred E3 ligases, conferring a large degree of substrate specificity to the ubiquitin cascade [11,25,166]. It follows that therapeutically targeting individual E3s, which selectively bind to the recognition sites of protein substrates, will achieve maximal target specificity, as compared to targeting any E1s or E2s. The inhibitor of the ubiquitin-like protein, neural precursor cell-expressed developmentally down-regulated 8 (NEDD8), MLN4924 (pevonedistat), was developed to inhibit Cullin-RING E3 ubiquitin ligases, the largest family of E3s, which require activation by E1/E2-mediated NEDDylation [165]. As a result of this mode of action inhibiting multiple E3 ligases, MLN4924 has broad target specificity. Although most of its adverse effects are mild or moderate, several higher-grade toxicities been reported, including febrile neutropenia, thrombocytopenia and elevated circulating levels of aspartate transaminase [165,166,177]. Notwithstanding, clinical trials of MLN4924 in combination with 5-azacytidine are underway in patients with acute myeloid leukaemia (AML) and are yielding promising initial results (Table 1) [177].

The F-box protein, β-TrCP, is involved in the signal-dependent recognition and degradative K48-linked ubiquitination of canonical IκBs and p100, as well as in the recognition and ubiquitination of a large group of other target substrates, including β-catenin, Snail, Emi1, Wee1, Cdc25A and Claspin [178,179]. From a theoretical standpoint at least, targeting NF-κB signalling via β-TrCP would represent a more specific and, possibly, safer alternative to proteasome inhibition [178,179], although a limitation of the approach would remain the accumulation of protein substrates outside the NF-κB pathway, such as β-catenin, which contributes to colorectal carcinogenesis [178,179]. Notwithstanding this caveat, small-molecule inhibitors of β-TrCP, such as GS143, have been developed and shown to inhibit signal-induced IκBα ubiquitination [180]. However, little information is available on the mode of action of this agent, although it seemingly involves an interaction with both β-TrCP and IκBα, or its further characterisation [165].

While proteasome inhibitors, such as bortezomib, can provide significant clinical benefit in multiple myeloma and a few other indications, these agents inhibit NF-κB and many other essential cellular pathways that rely on the proteasome function [6,162,166], and are therefore by no means specific for the NF-κB pathway. Moreover, proteasome inhibitors target these pathways in normal and cancer cells alike, thereby resulting in a low therapeutic index and significant dose-limiting toxicities [137,166,181,182]. Indeed, despite their indisputable commercial and clinical success, proteasome inhibitors as a therapy present several limitations, which remain largely unaddressed, including their broad cellular activities, dose-limiting side effects and the relatively rapid onset of secondary drug resistance [6,162,165,166]. In particular, treatment with bortezomib is often associated with peripheral neuropathy, which is a dose-limiting adverse effect, as well as trombocytopenia, neutropenia, nausea, diarrhea, and fatigue [162]. Furthermore, drug resistance is inevitable and generally develops within a year from the start of treatment [166]. While carfilzomib administration is associated with different side effects and the onset of peripheral neuropathy is significantly less frequent than with bortezomib, patients can nonetheless develop renal impairment and cardiovascular complications [162]. Likewise, treatment with Ixazomib can induce peripheral neuropathy, gastrointestinal adverse effects and skin rash [162].

Importantly, from a mechanistic standpoint, it is also unclear that the clinical response to proteasome inhibitors in patients with multiple myeloma and other B-cell malignancies results from the inhibition of NF-κB signalling, as it is becoming increasingly clear that the cellular accumulation of undigested proteins in these immunoglobulin-producing tumours can activate the unfolded protein response (UPR), thereby accelerating tumour-cell death [162]. Therefore, there remains a need for novel therapeutic strategies capable of selectively targeting the NF-κB pathway in these oncological indications [137,166,179]. While targeting upstream components of the UPP, such as NF-κB-activating E3 ligases, would be a preferable strategy than targeting the proteasome, as it could mitigate at least some of the limitations of proteasome inhibition, and, more broadly, represents a highly promising area for future drug discovery and development in oncology, this strategy would nevertheless be unlikely to ever yield a specific NF-κB-targeting agent [137,162,165,166].

### 3.4. Inhibitors of NF-κB Nuclear Activities

Once liberated from IκBs, NF-κB dimers migrate into the nucleus where they regulate gene expression. This activation step offers a significant opportunity for therapeutic intervention, since, at least from a theoretical standpoint, a number of nuclear activities of NF-κB could be targeted with drug agents, including the post-translational modification of NF-κB proteins and their ability to dimerise, translocate into the nucleus, bind to DNA and interact with chromatin components, coactivators and corepressors and other transcription factors [3,105]. While the drug development output in this area has been relatively limited, a few examples of candidate therapeutics targeting the nuclear translocation and DNA-binding activity of NF-κB complexes are worthy of consideration (Figure 3). In particular, several strategies have been developed to inhibit the NF-κB nuclear translocation, including small peptidomimetics, such as SN-50, which encompasses the NLS of p50 [3,105,183]. At high concentrations, SN-50 has been shown to saturate the transport machinery importing p50-containing dimers into the nucleus [3,105,183]. However, apart from the high peptide concentrations required to achieve this effect, a drawback of SN-50 is its non-specific inhibition of transcription factors other than NF-κB complexes, such as AP-1-family factors [3,105,183]. Additionally, dehydroxymethylepoxyquinomicin (DHMEQ), a derivative of the antibiotic epoxyquinomicin C, isolated from *Amycolatopsis*, has been shown to exhibit a potent and specific inhibitory activity on NF-κB nuclear import by means of its ability to directly bind to NF-κB dimers [3,105,183,184]. Studies in mouse models have demonstrated a therapeutic effect of DHMEQ in prostate carcinoma and an immunomodulatory effect in ovarian carcinoma. Despite these promising preclinical results, to our knowledge, no clinical trials have been initiated to evaluate DHMEQ in cancer patients [185,186,187].

Furthermore, sesquiterpene lactone (SL) compounds have been shown to inhibit the DNA-binding activity of RelA-containing NF-κB dimers by interacting with cysteine residue, C38, within RelA’s DNA-binding loop 1 (L1) [3,105]. By binding to homologous cysteine residues within p50 and c-Rel, certain SL compounds have been shown to have the additional capacity to inhibit NF-κB complexes containing these subunits [3,105]. Interestingly, some SLs, such as parthenolide, also have the ability to inhibit IKKβ [3,105]. However, while this dual effect of parthenolide on the NF-κB pathway has attracted some interest, the poor bioavailability of this agent has precluded any further drug development effort [142,188]. However, the search for a functionally equivalent compound has resulted in the generation of the amino acid-analogue, dimethylaminoparthenolide (DMAPT), exhibiting enhanced bioavailability [189]. A phase I clinical trial of DMAPT was initiated in 2009 in patients with AML, but was then suspended later in the same year [189,190]. Another class of agents designed to interfere with the NF-κB DNA-binding activity consists of decoy oligodeoxynucleotides, which can compete for binding to NF-κB complexes with κB DNA sites on specific gene promoters [2]. Most therapeutic decoy oligodeoxynucleotides were further modified to increase their in vivo stability, as well as their affinity for NF-κB complexes [3,105]. A clinical trial of the NF-κB decoy oligodeoxynucleotide, anesiva, was initiated in 2005 to test its efficacy as a local ointment for the treatment of atopic dermatitis, but its clinical use was subsequently discontinued in 2014 (NCT00125333, [190]).

### 3.5. Inhibitors of NF-κB Downstream Effectors

Since NF-κB induces transcriptional programmes that affect all hallmarks of cancer [79,191], an attractive alternative to therapeutically targeting NF-κB in malignant disease would be to inhibit the non-redundant, cancer cell-specific downstream effectors of the NF-κB oncogenic functions (Figure 3). Given that the insurmountable challenge with conventional NF-κB or IKKβ inhibitors has been to achieve cancer-cell specificity, due to the pleiotropic and ubiquitous functions of NF-κB [11], agents targeting these effectors, having functional restriction to cancer cells or their microenvironment, could provide safer and more selective anticancer therapeutics, lacking the dose-limiting toxicities of global NF-κB inhibitors.

Our group recently sought to obtain proof-of-concept for this principle in multiple myeloma, the paradigm of NF-κB-driven malignant diseases. Since a key pathogenetic function of NF-κB in multiple myeloma is to upregulate genes that block apoptosis and, despite its ubiquitous nature, NF-κB signalling induces highly tissue- and context-specific transcriptional programs [6,11,12,14,79,192], we targeted an essential downstream effector of this pathogenically critical activity of NF-κB, in order to achieve cancer cell-selective therapeutic specificity and thereby circumvent the limitations of conventional IKK/NF-κB-targeting drugs. Several years ago, we identified the immediate-early gene, growth arrest and DNA damage 45B (*GADD45B*), as a novel transcriptional target of NF-κB and effector of the NF-κB-dependent inhibitory activity on JNK signalling and apoptosis in response to TNF-α and other cues [193,194]. GADD45β is a member of the GADD45 family of proteins, also comprising GADD45α and GADD45γ, which play distinct roles in multiple cellular functions, including cell-cycle regulation, DNA repair, apoptosis, senescence and DNA demethylation [195,196,197]. Interesting, GADD45β is the only member of this family that is largely regulated downstream of NF-κB signalling [193,194]. A number of studies, including some from our own group, have demonstrated that GADD45β and other members of this family play many of their important biological roles by regulating the activity MAPK pathways, such as the JNK and p38 pathway [195,198,199]. Indeed, more recently, we have identified the complex formed by GADD45β and the JNK kinase, MKK7, as a functionally critical survival module downstream of NF-κB and novel therapeutic target in multiple myeloma [194,198,200,201,202,203]. As most normal cells do not constitutively express *GADD45B* [204], and, unlike mice lacking *RelA* or any *IKK* subunit [11], *Gadd45b^−/−^* mice are viable, fertile and die of old age [205,206], we reasoned that, in contrast to global NF-κB blockade, pharmacological GADD45β inhibition would be well tolerated in vivo.

We therefore developed the D-tripeptide inhibitor of the GADD45β/MKK7 complex, DTP3, which effectively disrupts this complex at nanomolar concentrations, in vitro, by binding to MKK7, and as a result, selectively kills multiple myeloma cells by inducing MKK7/JNK-dependent apoptosis, without toxicity to normal tissues [200,201]. We showed that, due to this target-selective mode of action, DTP3 displays an excellent cancer-cell selective specificity in multiple myeloma cell lines and primary cells from patients, in vitro, and eradicates multiple myeloma xenografts in mice, with excellent tolerability and no apparent side effects at the therapeutic doses [200,201]. The first-in-human phase I/IIa clinical study of DTP3 has recently been initiated in patients with refractory or relapsed multiple myeloma, and initial results from this study preliminarily demonstrate the clinical safety of this agent, alongside a cancer-selective pharmacodynamic response (Table 1).

Further investigations will be required to determine the potential clinical benefit resulting from this approach in multiple myeloma patients, as well as its potential side-effects, propensity to develop drug resistance, and therapeutic efficacy in combination with other agents [207,208]. Nevertheless, the available body of preclinical data and the encouraging initial clinical results preliminarily demonstrate that cancer-selective inhibition of the NF-κB pathway is possible and promises to provide an effective therapeutic strategy, with no preclusive toxicity, that could profoundly benefit patients with multiple myeloma and, potentially, other cancers where NF-κB is a driver of pathogenesis. Indeed, the same principle we developed of targeting an axis of the NF-κB pathway with cancer-restricted function, rather than NF-κB globally, could be similarly applied to also selectively inhibit NF-κB oncogenic functions beyond the suppression of cancer-cell apoptosis [200,201], such as functions in governing tumour-associated inflammation.

Several antiapoptotic genes, in addition to *GADD45B*, have been shown to be transcriptionally regulated by NF-κB, including those encoding various BCL-2-family members such as B-cell lymphoma-extra large (*Bcl-X_L_*), myeloid cell leukaemia sequence 1 (*MCL1*), *A1* (also known as BFL-1) and, at least in certain tissue contexts, B-cell lymphoma 2 (*BCL-2*) itself [191,209]. These proteins are also frequently deregulated in certain cancer types, often as a result of chromosomal translocations or other genetic abnormalities, and promote oncogenesis by means of their ability to suppress cancer-cell apoptosis [210,211,212,213]. BCL-2-family members have also been shown to contribute to NF-κB-dependent radio- and chemo-resistance in cancer [80]. Accordingly, the therapeutic inhibition of these proteins has also been pursued as a combination therapy with radiation and chemotherapeutic drugs to overcome resistance to these agents in recalcitrant forms of malignancy [214]. Congruently, certain NF-κB-dependent multiple myeloma cell lines express low levels of GADD45β and are completely refractory to DTP3-induced killing [200], confirming the existence of GADD45β-independent mechanisms for NF-κB-dependent survival in certain subtypes of multiple myeloma, and such mechanisms are certain to also exist in other types of malignancy. The BCL-2 family of proteins comprises an evolutionarily conserved group of 20 members, which share one or more of four so-called BCL-2 homology (BH) domains, referred to as BH1, BH2, BH3 and BH4, and are involved in either suppressing or promoting apoptosis [211,212,213]. The members of this family can be classified into three functionally and structurally distinct groups: prosurvival proteins such as BCL-X_L_, MCL1, A1/BFL-1 and BCL-2 itself; multidomain proapoptotic effectors such as BCL-2-associated X protein (BAX) and BCL-2 antagonist/killer (BAK); and BH3-only proteins, which convey apoptosis-initiating signals, such as BCL-2-interacting mediator of cell death (BIM), BH3 interacting-domain death agonist (BID), Bcl-2-associated death promoter (BAD), and p53 upregulated modulator of apoptosis (PUMA) [211,212,213]. Historically, the main strategy utilised to inhibit the antiapoptotic activity of BCL-2-family members has been to generate cell-permeable drug mimetics of the BH3 domains of proapoptotic BCL-2-like proteins, which neutralise prosurvival BCL-2 proteins by binding to their surface hydrophobic groove [211,212,213,215]. However, many of these agents have shown limited therapeutic activity as single agents and/or significant side-effects due to their low specificity [211,215]. A notable exception in this group of molecules has been ABT-199 (venetoclax, RG7601, GDC-0199), a potent and selective first-in-class BCL-2 inhibitor [211,213,215]. ABT-199 has demonstrated significant anticancer activity in various models of lymphoma and leukaemia, both in vitro and in vivo, and has entered clinical trials in patients with non-Hodgkin’s lymphoma, CLL, AML and T-ALL, both as a single agent and in combination with other drugs [211,216]. ABT-199 was subsequently granted breakthrough status designation by the FDA in 2016 for the treatment of patients with relapsed or refractory CLL with 17p deletion (Table 1).

## 4. Conclusions

Given the central role of aberrant NF-κB activation in the pathogenesis of the large majority of human diseases, the therapeutic targeting of the NF-κB pathway has been aggressively pursued by the pharmaceutical industry and academic laboratories for over two and a half decades. However, this goal has proven thus far an insurmountable challenge, due to the severe dose-limiting toxicities associated with the global suppression of NF-κB, resulting in the dismaying absence of a specific NF-κB or IKK inhibitor in the current anticancer armamentarium. Nevertheless, the past three decades, since NF-κB was first discovered in 1986, have witnessed tremendous advances in the understanding of the intricate signalling networks governing the NF-κB pathway and its multiple functions, and these efforts are now bearing some of the long-awaited fruits in the field of translational medicine, by pointing toward potential safer alternatives to therapeutic NF-κB inhibition. Undoubtedly, the targeting of upstream signalling mechanisms and protein-protein interactions governing the contextual and tissue-specific activation of NF-κB signalling and, at the opposite end of the spectrum, the non-redundant effectors of the diverse tissue-specific transcriptional programmes that NF-κB activates in order to exert its biological functions are amongst the most attractive strategies being developed to achieve contextual, cancer-cell selective therapeutic inhibition of the NF-κB pathway, and thereby circumvent the preclusive limitations of conventional NF-κB inhibitors. Perhaps, the most valuable lesson to be learnt from these initial therapeutic attempts is that the complexity of the intricate signalling networks governing the NF-κB regulation and function appears to hold the key to untangle the NF-κB therapeutic riddle and translate it into concrete clinical benefits. Indeed, while significant ground remains to be covered and the limited clinical successes obtained so far in select experimental clinical contexts have yet to transform into healthcare benefit for the broader patient population, the basic and translational knowledge unravelled by these studies on the biological complexity in the NF-κB pathway is providing tangible new opportunities for cancer-selective therapeutic intervention, which are certain to attract growing interest in the future and be further capitalised upon.

## Figures and Tables

**Table 1:** Selection of inhibitors of the NF-κB signalling pathway that are either in clinical development or have been clinically approved.

Compound	Molecular Target	Cancer Type	Ongoing Clinical Trials
***Upstream IKKs complex***			
Brentuximab (Vedotin)	CD30	HL, Anaplastic large cell lymphoma, etc.	NCT01657331, NCT02462538, NCT01807598, NCT02939014, NCT03007030, NCT02169505, NCT01900496, etc.
Ibrutinib (PCI-32765)	BTK	MCL, CLL, WM, DLBCL, FL, MM, and NSCLC, etc.	NCT02801578, NTC0275689, NCT02943473, NCT02321540, NCT02558816, NCT02420912, NCT02315768, NCT02451111, NCT02356458, etc.
IMO-8400	TLR 7, 8, and 9	WM, DLBCL	NCT02252146
LCL-161	cIAPs	Ovarian cancer, MM	NCT02649673, NCT02890069, NCT01955434
Birinapant (TL32711)	cIAPs	Solid tumours and high grade serous carcinomas	NCT02587962, NCT02756130
***Ubiquitin proteasome pathway***			
Bortezomib	Proteasome	AML, lymphoma, MDS, neuroblastoma, ALL, etc.	NCT02308280, NCT02535806, NCT01736943, NCT01534260, NCT02613598, NCT02356458, NCT01241708, NCT03016988, NCT02139397, NCT02237261, etc.
Carfizomib	Proteasome	MM, neuroendocrine cancer, NHL, DLBCL, MCL, FL, peripheral T-cell lymphoma, HL, T-cell NHL, solid tumours, leukaemia, etc.	NCT02302495, NCT02572492, NCT02318784, NCT02142530, NCT02867618, NCT01738594, NCT02512926, etc.
Ixazomib (MNL-9708)	Proteasome	Glioblastoma, MM, lymphoma, amyloidosis, solid tumours, B-cell lymphoma, lymphoma, etc.	NCT02630030, NCT02924272, NCT02942095, NCT02312258, NCT02477215, NCT02898259, etc.
MLN4924 (Pevonedistat)	NEDD8	AML, solid tumours, chronic myelomonocytic leukaemia, MDS	NCT01814826, NCT02782468, NCT02610777, NCT03009240, NCT03057366
***NF-κB target genes***			
DTP3	Gadd45β/MKK7	MM	MR/L005069/1
ABT-199	BCL-2	CLL, WM, MCL, AML, NHL, DLBCL, FL, MM, MDS, etc.	NCT02677324, NCT02471391, NCT02558816, NCT02203773, NCT02055820, NCT03136497, NCT03128879, NCT02427451, etc.

## Abbreviations

A1	BCL2A1—BCL2-related protein A1
A20	TNFAIP3 —Tumour necrosis factor α-induced protein 3
ABIN-1	A20 binding inhibitor of NF-κB
ADAP	Adhesion- and degranulation-promoting adaptor protein
ALCL	Anaplastic large-cell lymphoma
ALL	Acute lymphoblastic leukaemia
AML	Acute myeloid leukaemia
ARD	Ankyrin repeat domain
ATM	Ataxia telangiectasia mutated
BAD	Bcl-2-associated death promoter
BAFF	B-cell activating factor
BAK	BCL-2 antagonist/killer
BAX	BCL-2-associated X protein
BCL10	B-cell CLL/lymphoma 10
BCL-2	B-cell lymphoma 2
Bcl-XL	B-cell lymphoma-extra large
BCR	B-cell receptor
BH	BCL-2 homology
BID	BH3 interacting-domain death agonist
BIM	BCL-2-interacting mediator of cell death
BIR	Baculovirus IAP repeat
CAML	Calcium modulating ligand
CARD	Caspase recruitment domain
CBM	CARD11-BCL10-MALT1
CD40L	CD40 ligand
Cdc25A	Cell division cycle 25A
c-FLIP	Cellular FLICE/caspase8-like inhibitory protein
c-IAP	Cellular inhibitor of apoptosis
CLL	Chronic lymphocytic leukaemia
CYLD	Cylindromatosis lysine 63 deubiquitinase
DAMP	Damage-associated molecular patterns
DHMEQ	Dehydroxymethylepoxyquinomicin
DLBCL	Diffuse large B-cell lymphoma
DMAPT	Dimethylaminoparthenolide
DUB	Deubiquitination enzyme
Emi1-FBXO 5	F-box protein 5
EMT	Epithelial-mesenchymal transition
FBXW	F-box and WD repeat domain containing
FDA	US Food and Drug Administration
FL	Follicular lymphoma
GADD45β	Growth arrest and DNA damage inducible β
GBM	Glioblastoma multiforme
GRR	Glycine rich region
GSK3	Glycogen synthase kinase 3
HAT	Histone acetyltransferase
HDAC	Histone deacetylases
HL	Hodgkin’s lymphoma
HOIL-1L	RBCK1—RanBP-type and C3HC4-type zinc finger containing 1
HOIP	RNF31—Ring finger protein 31
IKK	IκB kinase
IL-1β	Interleukin 1 β
IL-1β	Interleukin 1β
IL-1βR	IL-1β receptor
IRAK	Interleukin-1 receptor-associated kinase
IκB	Nuclear factor of κ light polypeptide gene enhancer in B-cells inhibitor
JAK	Janus kinase
LPS	Lipopolysaccharide
LTβ	Lymphotoxin β
LUBAC	Linear ubiquitination assembly complex
MALT	Mucosa-associated lymphoid tissue
MALT1	Mucosa-associated lymphoid tissue lymphoma translocation protein 1
MAP	Mitogen activated protein
MAPKKK	Mitogen activated protein kinase kinase kinase
MAVS	Mitochondrial antiviral signalling protein
MCL	Mantle-cell lymphoma
MCL1	Myeloid cell leukaemia sequence 1
MDS	Myelodysplastic syndrome
MEKK	Mitogen activated protein kinase kinase
MM	Multiple myeloma
mTOR	Mechanistic target of rapamycin
MYD88	Myeloid differentiation primary response protein 88
NEDD8	Neural precursor cell-expressed developmentally down-regulated 8
NES	Nuclear export signal
NF-κB	Nuclear factor κ B (Nuclear Factor binding to the κ-Light-chain-enhancer B site)
NHL	Non-Hodgkin’s lymphoma
NIK	NF-κB-inducing kinase
NLR	NOD-like receptor
NLS	Nuclear localization signals
NOD	Nucleotide-binding oligomerisation domain
NSCLC	Non-Small Cell Lung Cancer
OTUD7B	OTU domain-containing protein 7B
PAMP	Pathogen-associated molecular pattern
PAR	Poly(ADP-ribose)
PARP-1	Poly(ADP-ribose)-polymerase-1
PDK	Phosphoinoisitide-dependent kinase
PDK1	3-Phosphoinositide-dependent protein kinase 1
PDLIM2	PDZ and LIM domain protein 2
PIASy	Protein inhibitor of activated STAT protein y
PIDD	p53-induced death domain protein
PKC	Protein kinase C
PP	Protein phosphatase
PTEN	Phosphatase and tensin homolog
PUMA	p53 upregulated modulator of apoptosis
RANKL	Receptor activator of NF-κB ligand
RAS	Rat sarcoma virus oncogene
RBX1	RING-box protein 1
RHD	Rel-homology Domain
RIP	Receptor interacting protein
RLH	RNA helicase
RLR	RIG-I-like receptor
SCF	SKP1-Cullin 1-F-box protein
Sharpin	SHANK-associated RH domain interacting protein
SL	Sesquiterpene lactone
SMAC	Second mitochondria-derived activator of caspases
SOCS-1	Suppressor of cytokine signalling-1
STAT	Signal transducer and activator of transcription
SUMO	Small ubiquitin-like modifiers
TAB	TAK1-associated binding protein
TACI	TNFRSF13B—TNF receptor superfamily member 13B
TAK1	Transforming growth factor β-activated kinase 1
T-ALL	T-cell acute lymphoblastic leukaemia
TBK1	TANK-binding kinase
TCR	T-cell receptor
TLR	Toll-like receptor
TME	Tumour microenvironment
TNF-R	TNF receptor
TNFSF	Tumour necrosis factor superfamily member
TNF-α	Tumour necrosis factor-α
TRADD	TNF receptor-associated death domain protein
TRAF	TNF receptor-associated factor
TRIM23	Tripartite motif protein 23
TWEAK	TNF-related weak inducer of apoptosis
UBA	Ubiquitin-activating enzyme, E1
UBC	Ubiquitin-conjugating, E2
Ubc/Uev	Ubiquitin-conjugating enzyme/ubiquitin E2 variant
UBD	Ubiquitin-binding domain
UPP	Ubiquitin-proteasome pathway
UPR	Unfolded protein response
USP	Ubiquitin-specific peptidase
WIP1	Wild-type p53-induced phosphatase 1
WM	Waldenström’s macroglobulinemia
WWOX	WW domain-containing oxidoreductase
βTrCP	β-transducin repeat-containing protein
